# Detection of olive oil adulteration with vegetable oils by ultra‐performance convergence chromatography‐quadrupole time‐of‐flight mass spectrometry (UPC^2^‐QTOF MS) coupled with multivariate data analysis based on the differences of triacylglycerol compositions

**DOI:** 10.1002/fsn3.1664

**Published:** 2020-05-25

**Authors:** Yinghua Luo, Boyan Gao, Yaqiong Zhang, Liangli (Lucy) Yu

**Affiliations:** ^1^ College of Food Science and Nutritional Engineering National Engineering Research Center for Fruit and Vegetable Processing Key Laboratory of Fruit and Vegetable Processing Ministry of Agriculture Engineering Research Centre for Fruit and Vegetable Processing Ministry of Education China Agricultural University Beijing China; ^2^ China‐Canada Joint Lab of Food Nutrition and Health (Beijing) Beijing Technology & Business University (BTBU) Beijing China; ^3^ Institute of Food and Nutraceutical Science School of Agriculture and Biology Shanghai Jiao Tong University Shanghai China; ^4^ Department of Nutrition and Food Science University of Maryland College Park MD USA

**Keywords:** multivariate data analysis, Olive oil adulteration, quadrupole time‐of‐flight mass spectrometry (QTOF MS), triacylglycerol, ultra‐performance convergence chromatography (UPC^2^)

## Abstract

Three different vegetable oils, including soybean, corn, and sunflower oils, were differentiated from olive oil by using ultra‐performance convergence chromatography coupled with quadrupole time‐of‐flight (UPC^2^‐QTOF MS) and multivariate data analysis based on their differences in triacylglycerol compositions. Then, olive oil was adulterated by adding these three vegetable oils in 1%, 0.75%, and 0.5% (v/v), and the adulterated olive oils were differentiated from the pure olive oils using the similar analytical strategies but different data processing approaches. After that, the representative markers in differentiating the adulterations were selected, and a mathematical model was created to detect the olive oil adulteration based on these specific markers. These results indicated that UPC^2^‐QTOF MS coupled with multivariate data analysis is a sensitive and accurate method in detecting olive oil adulteration, even in 0.5% adulteration level (v/v). This method could be applied in olive oil adulteration detection, and potentially beneficial to the oil industry.

## Graphical abstract

This manuscript reported the study of differentiating three different vegetable oils, including soybean oil, corn oil, and sunflower oil from olive oil by using UPC2‐QTOF MS and multivariate data analysis based on their differences in triacylglycerol compositions. After that, these three vegetable oils were adulterated to olive oil in 1%, 0.75%, and 0.5% (v/v), and the adulterated olive oil samples were differentiated from the pure olive oil using the similar analytical strategies but different data processing approaches. Then, the representative triacylglycerols in differentiating olive oil adulterations were selected and identified, and a mathematical model was created to detect the olive oil adulteration based on these specific triacylglycerols.

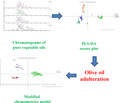

## 1 INTRODUCTION

Olive oil is one type of widely used vegetable oil in the Mediterranean diet. Nowadays, people all over the world tend to consume more olive oil due to its well‐known nutritional values, such as protecting low‐density lipoproteins (LDL) against oxidation (Fratianni et al., 2019; Vissers, Zock, & Katan, 2004), reducing potential cancer risk (Psaltopoulou, Kosti, Haidopoulos, Dimopoulos, & Panagiotakos, 2011), improving inflammatory disease (Parkinson & Keast, 2014; Schwingshackl, Christoph, & Hoffmann, 2015), and protecting the cardiovascular system (Hohmann et al., 2015; Piroddi et al., 2017). According to some previous literatures, the nutritional values of olive oil were mainly attributed to its high content of mono‐unsaturated fatty acid, especially high oleic acid content (Arlorio et al., 2010; Azadmard‐Damirchi, 2010; Mannina et al., 2009). Besides, the triacylglycerol (TAG) compositions of vegetable oils, especially the sn‐position of fatty acids in TAGs might also play very important roles in the nutritional values and physicochemical properties (Mateos et al., 2005; Yoshinaga et al., 2015). In 2015, Yoshinaga and colleagues demonstrated that sn‐position of bioactive unsaturated fatty acids, such as eicosapntemacnioc acid (EPA) and docosahexaenoic acid (DHA) in TAG could effectively determine the lipid synthesis and cholesterol metabolism in vivo. Considered the healthy benefit and wide existence (more than 98% of weight in olive oil are contributed by TAGs) of TAGs in olive oil, the chemical compositions of TAGs could be used to represent the profiles of olive oil.

Due to its high nutritional value and relatively low productivity, olive oil has always been recognized as one type of high economic value vegetable oil, and most of the olive oil adulterations were driven by economic interests. Adulterated olive oils would not only damage the economic profit of consumers, but also threat the public health. One of the most serious olive oil adulterations happened in Spain in 1981, where over 20,000 consumers were affected by the adulterated olive oil, more than 300 of them dead (Gelpí et al., 2002). To protect both the public health and economic interests, efficient and accurate analytical methods should be developed to monitor olive oil adulteration.

Till now, different detection methods for olive oil adulteration have been developed and can be divided into three major approaches: chemical methods (Azizian, Mossoba, Fardin‐Kia, Karunathilaka, & Kramer, 2016; Georgouli, Martinez Del Rincon, & Koidis, 2017; Jabeur, Drira, Rebai, & Bouaziz, 2017; Jabeur et al., 2014; Li et al., 2018; Sánchez‐Rodríguez, Sánchez‐López, Marinas, Urbano, & Caridad, 2019), biological methods (Santonico et al., 2015; Vietina, Agrimonti, & Marmiroli, 2013), sensory, and other methods (Biswas, Basumallick, Dasgupta, Ghosh, & Bandyopadhyay, 2016). Among them, chemical methods, including infrared spectrometry, Raman, LC‐MS, and GC‐MS, have been widely applied for olive oil adulteration detection. In 2017, Georgouli and colleagues reported a novel continuous statistical modeling for detecting extra virgin olive oil adulterated with hazelnut oil using Fourier transform infrared (FT‐IR) and Raman spectrometry. The results in this study represented that FT‐IR and Raman could be used to detect olive oil adulterated by small amount of hazelnut oil. In 2013, Yang and others reported the application of GC‐MS coupled with chemometrics to detect olive oil adulteration. Based on their results, data collected by GC‐MS, including the amounts of 22 fatty acids and 6 other parameters (the ratio of linoleic/linolenic acid, oleic/linoleic acid, total saturated fatty acids (SFAs), polyunsaturated fatty acids (PUFAs), monounsaturated fatty acids (MUFAs), and MUFAs/PUFAs), together with PLS‐DA could be used for the detection of olive oil adulteration with other vegetable oils in 1% detection limit and 90% prediction ability. High‐resolution mass spectrometry such as matrix‐assisted laser‐desorption/ionization time‐of‐flight (MALDI‐TOF) MS could also be used for the detection of olive oil adulterated by hazelnut oil (Calvano, Ceglie, D'Accolti, & Zambonin, 2014; De Ceglie, Calvano, & Zambonin, 2014). By detecting the possible hazelnut proteins or the phospholipids in the olive oil samples, MALDI‐TOF MS could detect 1% of hazelnut oil adulterated in olive oil (Calvano et al., 2014; De Ceglie et al., 2014). Although these chemical approaches could effectively detect olive oil adulteration, there are still some limitations of these methods. For instance, most of the GC analytical methods need sample derivatization, which are relatively high time‐cost and potential toxic to the researchers. Besides, previous chemical approaches could only supply limited chemical information about adulterants, or just appropriate for specific adulterant. All these limitations made it necessary to develop novel approaches to differentiate olive oil adulteration more accurately and effectively.

An improved supercritical fluid chromatography (SFC), the ultra‐performance convergence chromatography (UPC^2^) have been applied coupled with quadrupole time‐of‐flight mass spectrometry (QTOF MS) in detecting triacylglycerols from milk fat, vegetable oil, and other samples (Gao et al., 2017; Zhou et al., 2014). As an effective and environmental‐friendly analytical approach, UPC^2^‐QTOF MS is especially suitable in separating and identifying triacylglycerols from food matrixes. In this study, olive oil adulterated by other vegetable oils, including soybean oil, corn oil, and sunflower oil in different ratios, was detected using UPC^2^‐QTOF MS system; data were processed using multivariate data analysis. The aim of this study was to develop a whole solution, including an appropriate analytical method together with data processing approach to detect olive oil adulteration with trace amount of vegetable oils accurately and effectively.

## 2 MATERIALS AND METHODS

### 2.1 Materials and reagents

A total of 46 different brands of commercial vegetable oils, including 16 olive oils, 10 soybean oils, 10 corn oils, and 10 sunflower oils, were purchased from local groceries. Similar amount of each olive oil was mixed together as an olive oil representative. Soybean, corn, and sunflower oil representatives were processed in the same way. After that, soybean, corn, and sunflower oil representatives were individually mixed with olive oil representative in 1%, 0.75%, and 0.5% (v/v) to make the adulterated olive oil samples, respectively. LC‐MS grade acetonitrile, ammonium formate, methanol, and isopropanol were purchased from Sigma‐Aldrich (St. Louis, MO, USA). LC‐MS grade water was obtained from a Milli‐Q 10 ultra‐pure water system (Billerica, MA, USA). All the other reagents were analytical grade and used without further purification.

### 2.2 Sample preparation

All the pure oil samples were prepared based on our previous study.^24^ Briefly, 10 μL of each oil sample was vortexed with 990 μL of acetonitrile/methanol/isopropanol (10:9:1, v/v/v) for 20 s. The mixture was centrifuged at 376 g for 5 min to remove the supernatant. Then, the residue was dissolved with 990 μL of isopropanol before the UPC^2^‐QTOF MS analysis. All the oil samples were analyzed in triplicates.

For the adulterated oil samples, all the olive oil samples were mixed in the same volume to prepare the standard olive oil sample, similar progresses were performed for soybean, corn, and sunflower oils to prepare standard soybean, corn, and sunflower oil samples. Then, 100, 75, and 50 μl of standard soybean oil were mixed with 9.9, 9.925, 9.95 ml of standard olive oil to prepare 1%, 0.75%, and 0.5% soybean oil adulterated olive oil samples, respectively. Similar approaches were practiced to prepare corn oil and sunflower oil adulterated olive oil samples. After that, the adulterated oils were extracted using similar procedures as used in pure oil extraction.

### 2.3 UPC^2^‐QTOF MS conditions

A Waters Acquity UPC^2^ (Waters, Milford, MA, USA) coupled with Water Xevo‐G2 QTOF MS system was utilized in this study. An UPC^2^ HSS C18 column (150 mm × 3.0 mm i.d.; 1.7 μm) was used with oven temperature at 30°C. The mobile phase A was CO_2_, and the mobile phase B was methanol. A gradient elution started at 1% B, increased linearly to 1.8% B at 5 min, 2% B at 12 min, 3% at 15 min, 30% at 16 min, and 35% at 19 min and went back to the initial ratio and equilibrium for the next injection. The injection volume was 2.0 μl, and the flow rate was 1.6 ml/min with the back‐pressure set at 1,800 psi. A Waters 1,525 single pump was selected as the compensated pump, using 0.1% ammonium formate in methanol as the solvent at a flow rate of 0.3 ml/min.

For the MS condition, an electrospray ionization (ESI) positive mode was selected in this study. The data acquisition range was m/z 100–1,200 Da for both MS^1^ and MS^2^ modes. Leucine encephalin ([M + H]^+^ m/z 556.2,771) was used as the lock mass, with the infusion rate at 5 μl/min. The capillary, sampling cone, and source offset voltage were 3.0 kV, 50.0 V, and 80.0 V, respectively. The source and desolvation temperatures were 120°C and 500°C, respectively. The collision gas was argon with a flow rate of 150 L/h, and the desolvation gas was nitrogen at the flow rate of 800 L/h. *MSE* scan mode was selected in this study, for which both a low‐collision energy scan mode (collision energy at 6 eV) for MS^1^ and a high‐collision energy scan mode (collision energy at 35 eV) for MS^2^ were programmed. Data were collected with Waters Masslynx v4.1 software.

### 2.4 Data processing

Data were processed with Waters Nonlinear™ Progenesis QI (Waters, Milford, MA, USA) software and analyzed by SIMCA embedded in Waters Ezinfo software. All the raw data were imported to Progenesis QI, normalized, and aligned all the chromatograms by comparing with the selected reference chromatograms, grouping, and exported to Ezinfo. After that, both PCA and PLS‐DA were used to analyze the data. In data processing models, all the samples were autoscaled before analysis.

## 3 RESULTS AND DISCUSSION

This study aims to investigate the effects of UPC^2^‐QTOF MS coupled with chemometrics in differentiating olive oil adulterated with other commercial vegetable oils. So the first step is to distinguish olive oil from vegetable oils, including soybean, corn, and sunflower oils. After that, olive oils adulterated with different ratios of other vegetable oils will be analyzed to determine the efficiency of this approach, representative markers from this data model could be selected. And finally, 10 most representative markers will be used to create the new model, which could be used to differentiate olive oil adulteration effectively and accurately.

### 3.1 Differentiating pure soybean, corn, and sunflower oils from olive oil

**Figure 1:**
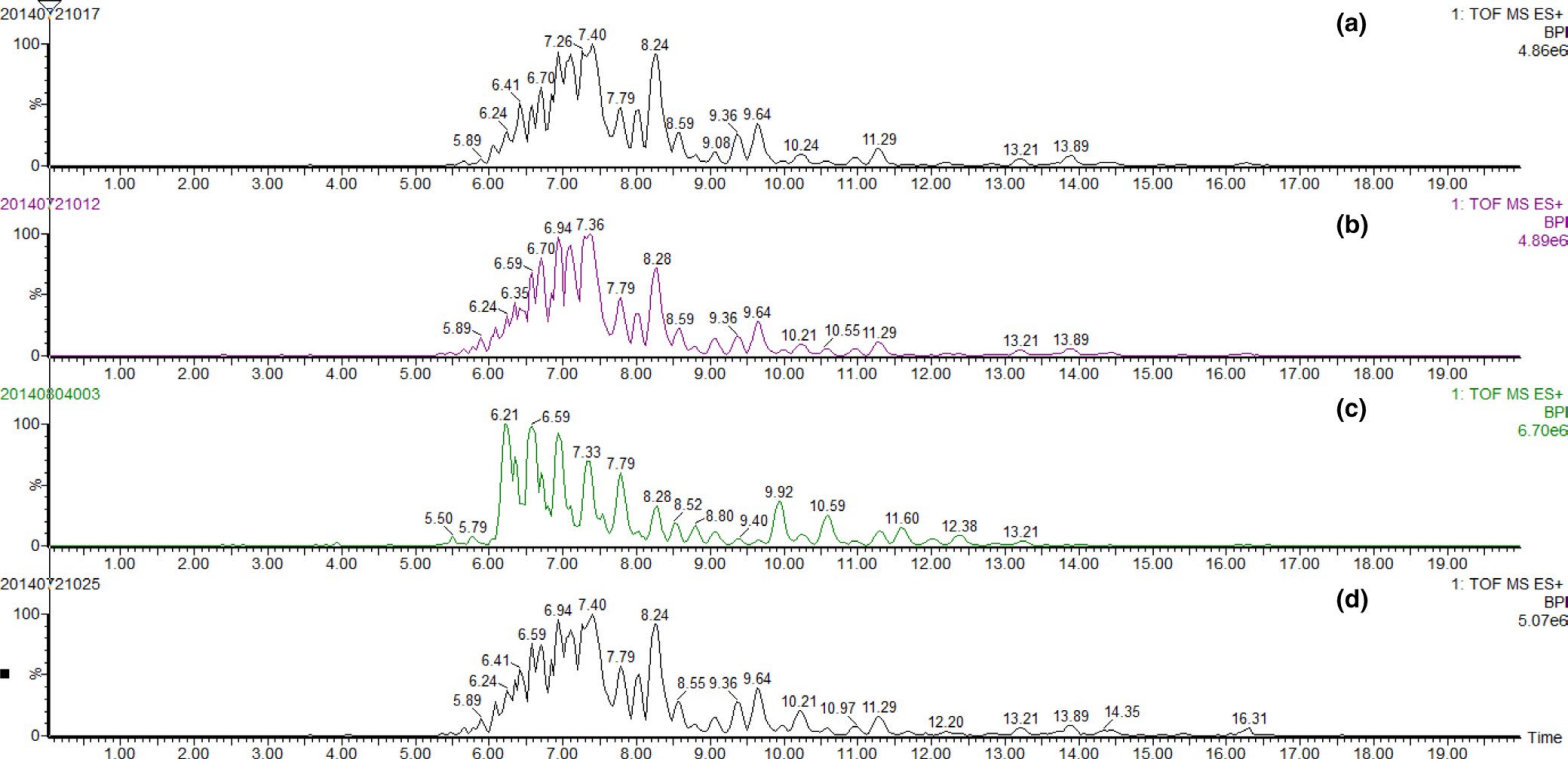
Typical UPC^2^‐QTOF MS chromatograms of triacylglycerols from pure (a) soybean oil, (b) corn oil, (c) sunflower oil, and (d) olive oil samples

Typical UPC^2^‐QTOF MS chromatograms for the triacylglycerols profiles of pure soybean, corn, sunflower, and olive oil representative samples were showed in Figure 1. The triacylglycerol compositions of these vegetables oils were reported in our previous studies.^24^ Briefly, triacylglycerols LLL, PLL, BLL, and MLL could be detected in soybean, corn, and sunflower oils, but not in olive oil; on the other hand, triacylglycerols PoOPo, OPoO, and GOO could be detected only in olive oil sample. Besides, the relative amount of OOO was much greater in olive oils (more than 20% in the total amount of olive oil triacylglycerols), compared with that in other vegetable oils (less than 10%). These results could also be confirmed by previous studies that oleic acid was the most abundant fatty acid in olive oil fatty acids (Jabeur el al., 2014; Yang, Ferro, Cavaco, & Liang, 2013). Therefore, the compositions of these different triacylglycerols could be used as the target various in differentiating the olive oils from soybean, corn, or sunflower oils.

**Figure 2:**
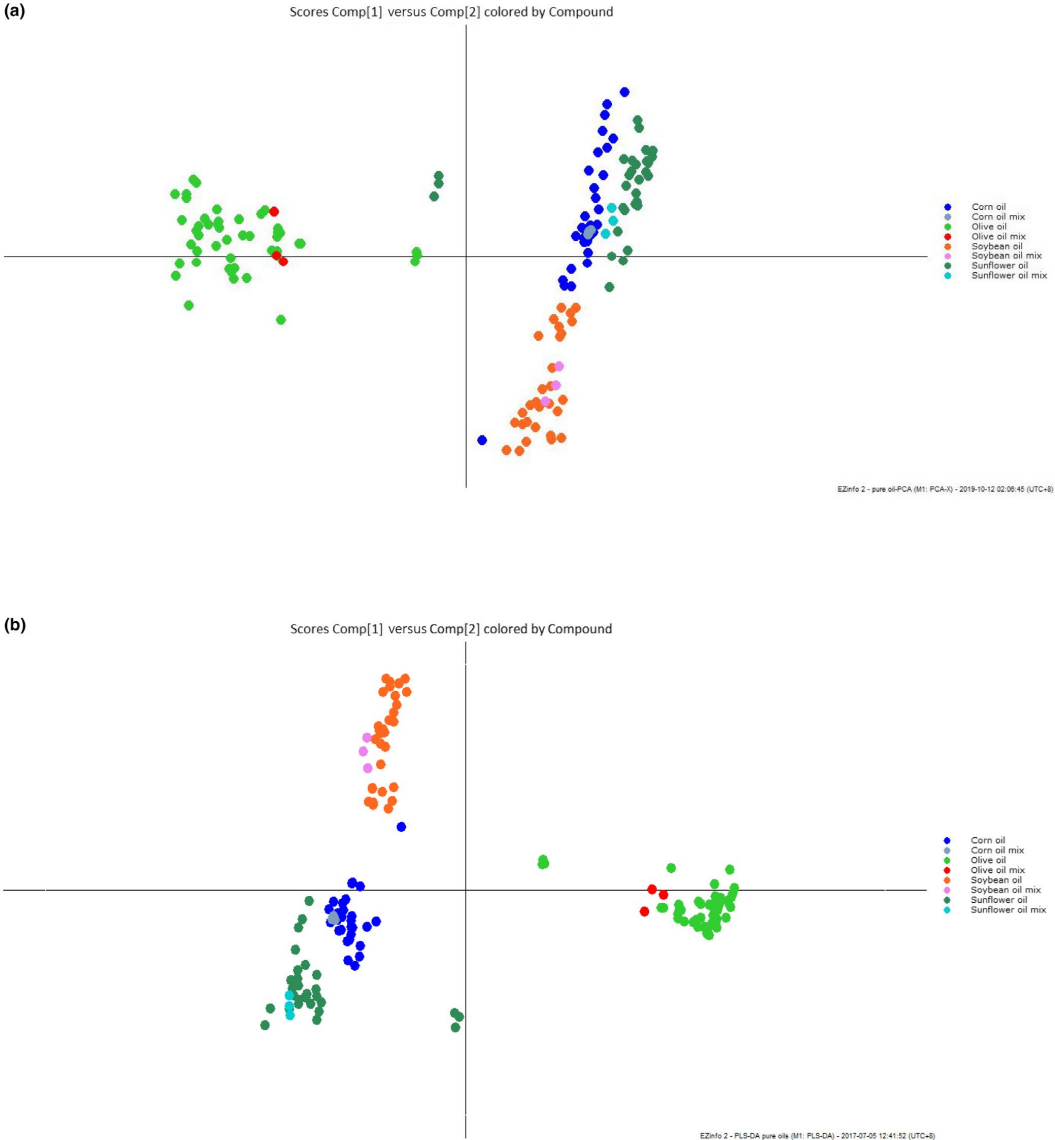
(a) PCA scores plot and (b) PLS‐DA scores plot for pure vegetable oils

Unbiased data processing method, principle component analysis (PCA) was used firstly in differentiating pure olive oil samples from soybean, corn, and sunflower oils by analyzing their triacylglycerol compositions. As the results, PCA could effectively differentiate pure olive oil from other three pure vegetable oils with unbiased data processing method (Figure 2a), which could be confirmed by previous review article and our previous study that the triacylglycerol compositions of pure olive oils are quite different from pure soybean, corn, or sunflower oils and easy to be differentiate (Gao et al., 2017; Indelicato et al., 2017). On the other hand, results from PCA also represented that the triacylglycerol compositions of corn, soybean, and sunflower oil samples were almost similar and could not be differentiate. In order to better differentiate all the oil samples, other biased data processing methods might be utilized to perform better results.

Then, the obtained triacylglycerol compositions of pure soybean, corn, sunflower, and olive oils were processed by a biased data analyzing approach, partial least squares‐discriminant analysis (PLS‐DA). As showed in Figure 2b, the PLS‐DA scores plot indicated that all the olive oil samples were clustered tightly on the right side. All the soybean oil samples were clustered on the top left side of scores plot, while corn oil samples and sunflower oil samples were on the bottom left side. These results indicated that the combination of UPC^2^‐QTOF MS and PLS‐DA could effectively differentiate pure olive oil from soybean, corn, or sunflower oil samples through their triacylglycerol compositions. Results from the loading plot of this model represented that various with m/z at 479.4086 ([M + H]^+^, C_30_H_55_O_4_) and 451.3776 ([M + H]^+^, C_28_H_51_O_4_) contributed most to the olive oils, which should be the fragment ions of typical triacylglycerols in olive oils. On the other hand, m/z at 581.4567 ([M + H]^+^, C_38_H_61_O_4_), 317.2480 ([M + H]^+^, C_21_H_33_O_2_), and 653.4470 ([M + H]^+^, C_44_H_61_O_4_) contribute to soybean oils; whereas m/z 615.4984 ([M + H]^+^, C_39_H_67_O_5_), and 701.6062 ([M + H]^+^, C_45_H_81_O_5_) contribute most to both corn and sunflower oils. These results above proved that UPC^2^‐QTOF MS coupled with chemometrics could differentiate pure olive oils from other pure vegetable oils effectively. And the next step of this study is to differentiate pure olive oils adulterated with different ratios of other vegetable oils.

### 3.2 Differentiating pure olive oil and olive oil adulterated with other vegetable oils

**Figure 3:**
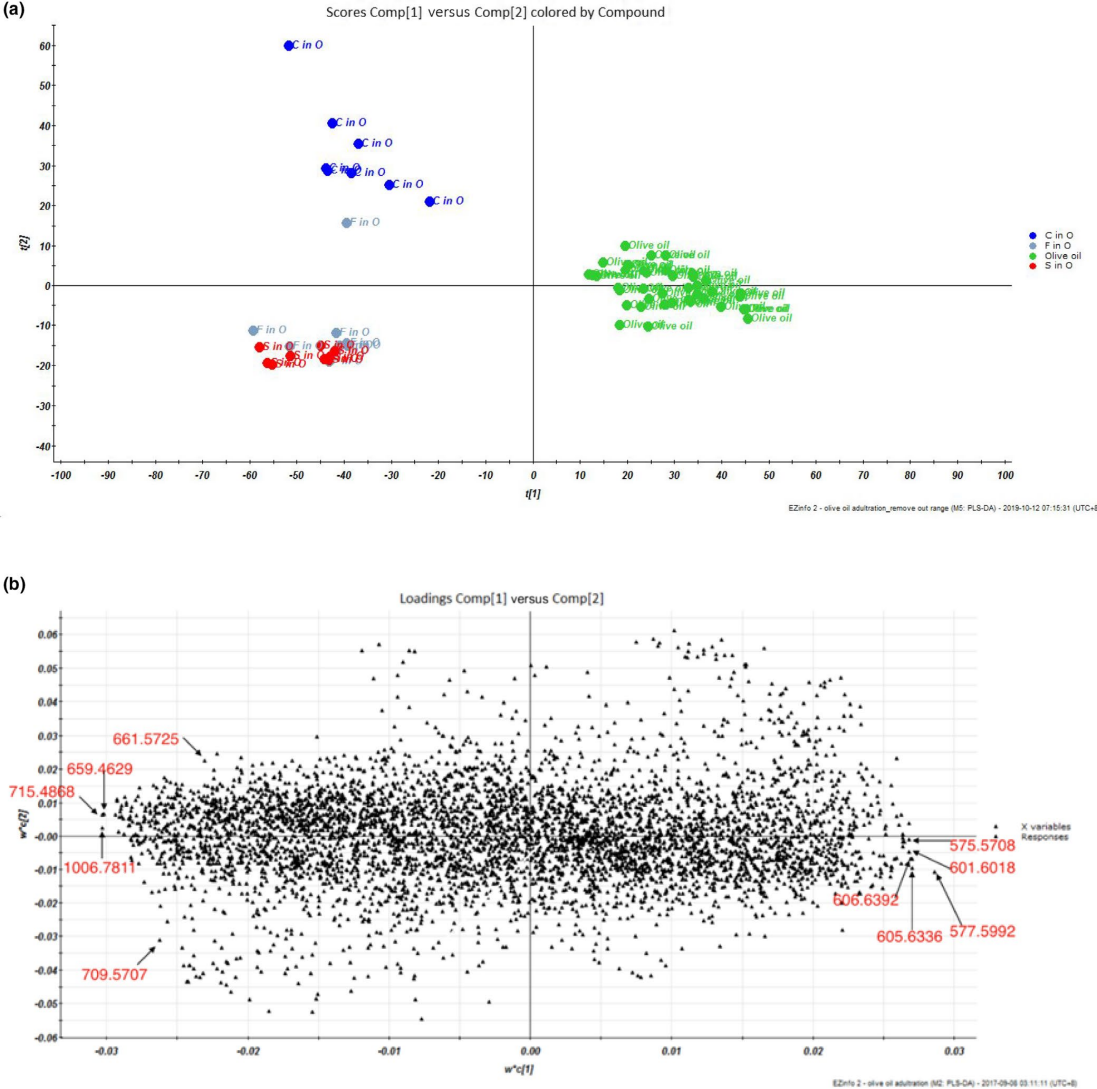
PLS‐DA (a) scores plot and (b) loading plot of pure olive oil and olive oil adulterated by different ratios of soybean, corn, or sunflower oils, based on all the triacylglycerol information. S in O represented soybean oil adulterated in olive oil, F in O represented sunflower oil adulterated in olive oil, C in O represented corn oil adulterated in olive oil

The pure olive oil adulterated with 1%, 0.75%, and 0.5% (v/v) of soybean oil, corn oil, or sunflower oil samples was analyzed with UPC^2^‐Q TOF MS system using the similar approaches described before. There was no visual difference between the chromatograms for the TAG profiles of pure olive oil and the adulterated olive oils (data not shown). While analyzed by PLS‐DA, the adulterated olive oils could be easily differentiated from the pure olive oil based on their different TAG compositions, even at 0.5% adulteration level (Figure 3a). The pure olive oil was clustered on the right side of the scores plot, whereas all the adulterated olive oils were on the left side. Among all the adulterated olive oils, olive oil adulterated with corn oil was on the top left side of scores plot and olive oil adulterated with soybean or sunflower oils was on the bottom left side of scores plot. These results represented that the UPC^2^‐QTOF MS together with PLS‐DA could be used to differentiate pure olive oil from olive oil adulterated with soybean, corn, or sunflower oils in a relatively low adulteration ratio.

Previous studies also reported the detection approaches in differentiating the purity of vegetable oils by using different analytical approaches. Among all those results, gas chromatography (GC) or GC‐MS was the most widely used method, and the working mechanism of GC/GC‐MS is to hydrolyze and methylate the triacylglycerol into free fatty acids, then analyze the compositions of free fatty acids in different oil samples to differentiate the triacylglycerol compositions of oils (Strashnov et al., 2019; Tian, Zeng, Zheng, Chiu, & Liu, 2019). The benefits of GC‐based methods are their stability and repeatability, but GC could not provide specific chemical information about the compositions of triacylglycerols directly. Other analytical methods, including Raman or FT‐IR, LC‐MS also performed remarkable abilities in differentiating vegetable oils, but no study before reported the results about detecting oil adulteration in less than 1% (Baeten et al., 2005; Farley et al., 2017; Fasciotti & Pereira Netto, 2010).

PLS‐DA loading plot of this result indicated the contributions of triacylglycerol ions or triacylglycerol fragment ions to differentiating the pure olive oils from the adulterated olive oils. In Figure 3b, the fragment ions at m/z 575.5708, 577.5992, 601.6018, 605.6336, and 606.6392 contribute most to the pure olive oils, while peak with greater natural abundant of ions at m/z 659.4629, 661.5725, 709.5707, 715.4868, and 1006.7811 are more likely contributed to the adulterated olive oils. In all these typical markers, ion at m/z 575.5708 could be identified as P‐L fragment ion, and ion at m/z 577.5992 might be the fragment ion of P‐O. These results indicated that some ions contributed more to differentiating the olive oil adulteration, and a novel mathematical model could be created based on these specific ions. These ions were selected based on their contributions in the loading plot, as well as the importance of their identifications as the triacylglycerols or fragments of triacylglycerols. Finally, a total of 10 ions were manually selected to create the model and aimed to differentiate the olive oil adulteration, including m/z 575.5708, 577.5992, 601.6018, 605.6336, 606.6392, 659.4629, 661.5725, 709.5707, 715.4868, and 1006.7811. These 10 ions were selected based on there are the markers contributed most in differentiating two groups of samples in the loading plots (Figure 3b).

### 3.3 Differentiating pure olive oil from adulterated olive oils based on 10 selected triacylglycerol and/or fragment ions

**Figure 4:**
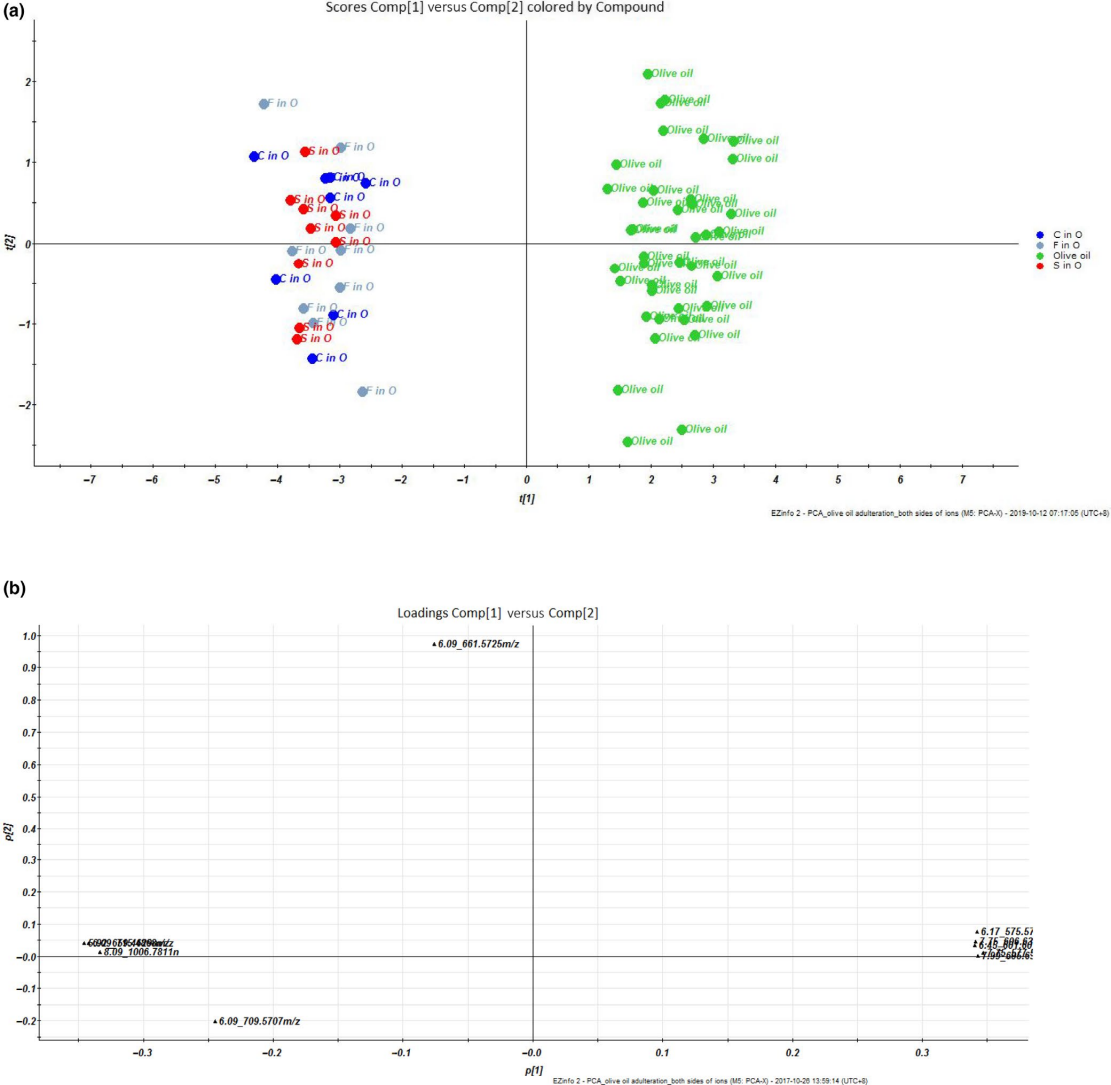
PCA (a) scores plot and (b) loading plot of pure olive oil and olive oil adulterated by different ratios of soybean, corn, or sunflower oils, based on 10 selected triacylglycerols. S in O represented soybean oil adulterated in olive oil, F in O represented sunflower oil adulterated in olive oil, C in O represented corn oil adulterated in olive oil

The principal component analysis (PCA) about the pure olive oils and adulterated olive oils was analyzed based on 10 ions selected above. In Figure 4a, all the pure olive oils were on the right side, and all the adulterated olive oils were on the left side. All the oil samples were included in the 95% confidence interval. These results represented that by using 10 selected ions, the adulterated olive oils could be differentiated from the pure olive oils using PCA, even in 0.5% adulteration ratio. In the loading plot of PCA results (Figure 4b), ions at m/z 575.5708, 577.5992, 601.6018, 605.6336, and 606.6392 contributed more in pure olive oils, whereas ions at m/z 659.4629, 661.5725, 709.5707, 715.4868, and 1006.7811 contributed more in the adulterated olive oils.

To sum up, this study reported a novel application of UPC^2^‐QTOF MS together with chemometrics in differentiating olive oil adulteration. By analyzing the triacylglycerol compositions, olive oils adulterated with soybean, corn, or sunflower oils at 0.5% level could be differentiated from pure olive oil based on their different triacylglycerol ions or fragment ions information. Although such a low ratio of adulteration might not happen in the real food industry, this model could still be recognized as a novel tool in determining olive oil adulteration or the purity of olive oil samples effectively and accurately. In addition, 10 representative marker ions were selected to create the model, which could be used to differentiate olive oil adulteration rapidly and accurately. The application of this method could provide not only the triacylglycerol compositions information about vegetable oils, but also the approaches of detecting olive oil adulterated with other vegetable oils in very low ratios effectively and accurately.

## CONFLICT OF INTEREST

None declared.

## ETHICAL APPROVAL

This study does not involve any human or animal testing.
